# Composite lymphoma of concurrent T zone lymphoma and large cell B cell lymphoma in a dog

**DOI:** 10.1186/s12917-019-2154-8

**Published:** 2019-11-16

**Authors:** Arata Matsuyama, Dorothee Bienzle, Danielle Richardson, Nariman Deravi, Mei-Hua Hwang, Nikos Darzentas, Stefan M. Keller

**Affiliations:** 1 Departments of Biomedical Sciences, University of Guelph, Guelph, ON N1G 2W1 Canada; 2 Departments of Pathobiology, University of Guelph, Guelph, ON N1G 2W1 Canada; 3 Departments of Clinical Studies, University of Guelph, Guelph, ON N1G 2W1 Canada; 4 Present address: Idexx Laboratories, 1345 Denison St., Markham, ON L3R 5V2 Canada; 5 Department of Internal Medicine II, University Hospital Schleswig-Holstein, Kiel, Germany/Central European Institute of Technology, Masaryk University, Brno, Czech Republic; 6 Present address: Department of Pathology, Microbiology, Immunology, University of California, Davis, USA

**Keywords:** Canine, Clonality, Dog, Lymphoma, Lymphosarcoma, Antigen receptor gene rearrangement, PARR, Composite lymphoma

## Abstract

**Background:**

Evolution of indolent to aggressive lymphoma has been described in dogs but is difficult to distinguish from the de novo development of a second, clonally distinct lymphoma. Differentiation of these scenarios can be aided by next generation sequencing (NGS)-based assessment of clonality of lymphocyte antigen receptor genes.

**Case presentation:**

An 8-year-old male intact Mastiff presented with generalized lymphadenomegaly was diagnosed with nodal T zone lymphoma (TZL) based on cytology, histopathology, immunohistochemistry and flow cytometry. Thirteen months later, the dog re-presented with progressive lymphadenomegaly, and based on cytology and flow cytometry, a large B cell lymphoma (LBCL) was diagnosed. Sequencing-based clonality testing confirmed the de novo development of a LBCL and the persistence of a TZL.

**Conclusions:**

The occurrence of two distinct lymphoid neoplasms should be considered if patient features and tumor cytomorphology or immunophenotype differ among sequential samples. Sequencing-based clonality testing may provide conclusive evidence of two concurrent and distinct clonal lymphocyte populations, termed most appropriately “composite lymphoma”.

## Background

Lymphoma is the most common hematopoietic neoplasm in dogs and is due to clonal proliferation of lymphocytes [1]. The clinical features of lymphoma vary widely, and range from slowly progressive indolent forms with modest tumor burden to rapidly progressive forms with large tumor burden and profound general illness [2, 3]. Attempts have been made to predict the clinical progression of lymphoma for accurate prognosis and appropriate therapy. Adaptation of the World Health Organization’s classification scheme for lymphoma in humans to samples of lymphoma from dogs identified six major entities [4]. Among these were diffuse large B cell lymphoma (DLBCL) and T zone lymphoma (TZL), which are defined by tumor architecture, histomorphology and immunophenotype [4]. Diffuse large B cell lymphoma is characterized by high mitotic count, expression of the B cell antigens CD79a, CD20 and/or CD21, and rapid progression [5]. TZL is typically associated with slowly enlarging lymph nodes, low-grade lymphocytosis, and expression of T cell antigens such as CD3, CD5, and/or CD4 or CD8 [2, 6].

Approaches to diagnose and classify lymphoma include cytology, histopathology, immunohistochemistry, flow cytometry, and clonality testing. Although histopathology combined with immunohistochemistry is generally sufficient for diagnosis, obtaining a tissue biopsy requires sedation or anesthesia. Aspirating lymph nodes is less invasive, and since most lymphomas in dogs have diffuse architecture, representative samples for diagnosis are readily obtained. Aspirated samples are also suitable for flow cytometric characterization and, therefore, prognostication [7]. In cases of lymphomas with mixed cell composition, or unusual histomorphology, additional testing such as clonality assessment, may be required. Clonality testing, in veterinary medicine also known as polymerase chain reaction (PCR) for antigen receptor gene rearrangement (PARR), detects rearranged antigen receptor genes by PCR-based amplification and evaluation of amplicon sizes by high-resolution electrophoretic analysis [8]. Both flow cytometry and clonality testing can be used for confirmation or classification of canine lymphoma, but infidelity in lymphocyte antigen expression and clonal rearrangements has also been identified in non-lymphoid neoplasms of dogs [9]. Furthermore, robust data concerning sensitivity and specificity of either assay are limited. With next generation sequencing (NGS)-based clonality testing, thousands to millions of lymphocyte antigen receptor gene sequences amplified in a single run are analyzed quantitatively. This technology, while more complex and expensive, circumvents certain shortcomings of conventional clonality assays such as interpretative subjectivity of electrophoretic peaks and false positive results from presence of multiple clones of similar size. Since NGS-based clonality testing identifies clones by sequence, this methodology allows monitoring of patient-specific tumor clones during and after therapy, an application known as minimal residual disease (MRD) monitoring [10].

This is the first report of a dog with concurrent TZL and LBCL diagnosed using multiple diagnostic approaches. Clinical features and tumor cytomorphology suggested emergence of a high-grade lymphoma in a patient with a pre-existing indolent TZL. Flow cytometry confirmed the initial subtype of TZL and subsequent LBCL, and NGS-based clonality testing identified two distinct clones within one affected lymph node.

## Case presentation

An 8-year-old male intact Mastiff presented to a veterinary specialty hospital with an acute onset of head shaking. Magnetic resonance imaging (MRI) of the head revealed an intra-axial lesion within the right pyriform lobe. The lesion was T2/FLAIR hyperintense, T1 hypointense, and non-contrast enhancing with a mild mass effect, consistent with a primary neoplasm such as glioma. The dog was subsequently referred to the Ontario Veterinary College Animal Cancer Centre, and definitive radiation therapy consisting of 18 fractions was administered over 4 weeks at a total dose of 45 Gray.

**Fig. 1:**
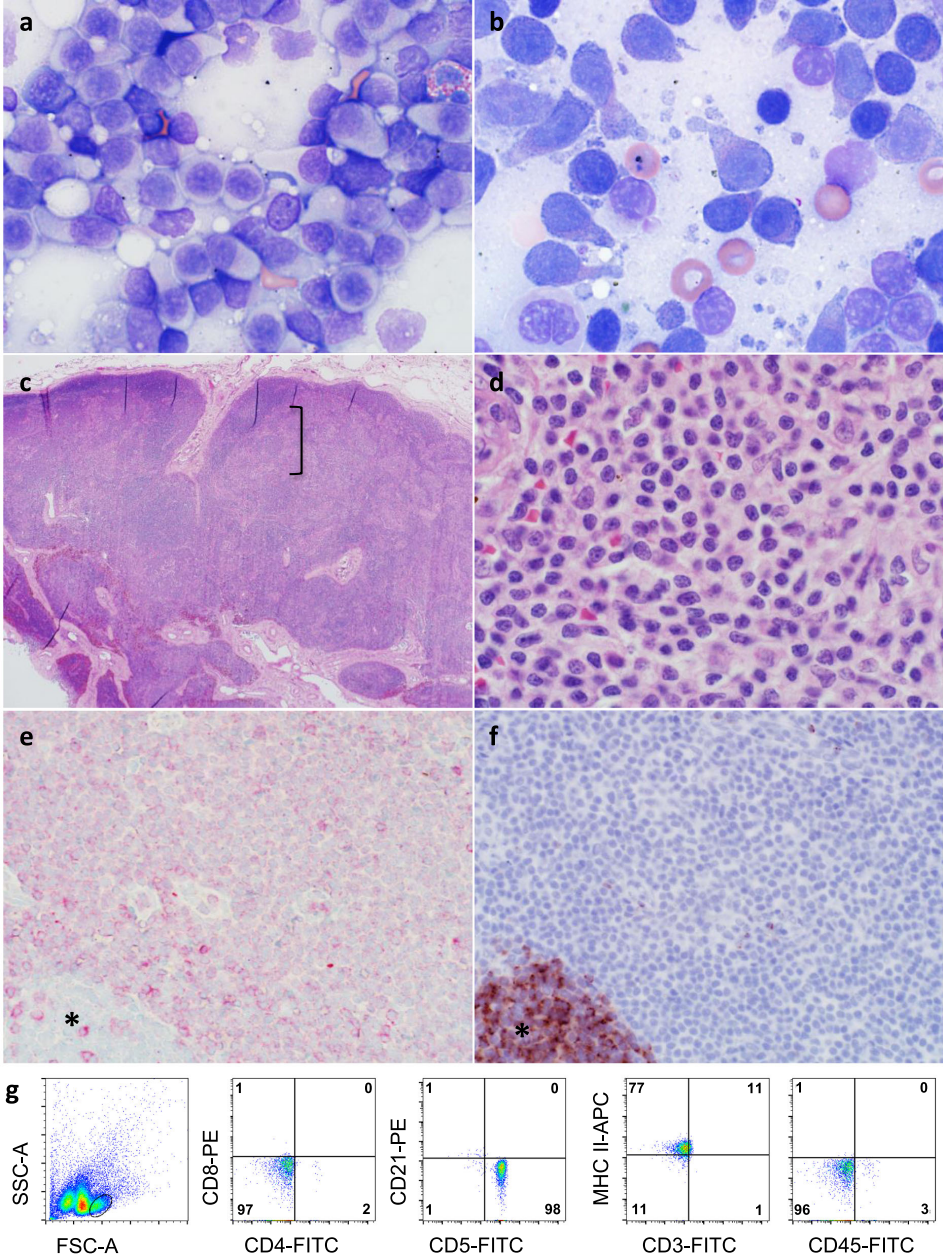
**a** An aspirate of an enlarged popliteal lymph node consists of a predominant population of small lymphocytes that have a moderate amount of pale cytoplasm. **b** At higher magnification frequent cytoplasmic fragments and “hand-mirror” appearance of cells are evident. **c** On histopathology, cortico-medullary distinction of the lymph node is distorted by an expansion of paracortical lymphocytes (bracket). **d** At higher magnification, mitotic figures are absent. **e** With immunohistochemistry, there is faint cytoplasmic and membranous reactivity for CD3 on the neoplastic cells and absent reactivity on a remnant follicle (*), while in **f** reactivity for CD79a is absent on neoplastic cells but prominent on a remnant follicle (*). **g** On flow cytometry, small lymphocytes are uniformly positive for CD5 and MHC II, and negative for CD4, CD8, CD21 and CD45. There is dim detection of CD3. These findings are consistent with TZL

At a re-check appointment 7 months after completion of radiation therapy, the dog was free of neurological signs while receiving solely oral phenobarbital (120 mg, q12h) but physical examination revealed mild generalized lymphadenomegaly. Results of a complete blood cell count (CBC) and serum biochemistry analysis were unremarkable. Fine needle aspirates of three peripheral lymph nodes yielded slides with a relatively uniform population of medium size lymphocytes with pale cytoplasm, central or slightly eccentric nuclei and frequent cytoplasmic projections. The findings were interpreted as lymphoma, and the cytomorphology was consistent with TZL [11] (Fig. 1a and b). Histopathology of an excisional biopsy of the left popliteal lymph node showed a paracortical expansion in between “fading” follicles with monotypic lymphocytes arranged in sheets with minimal anisocytosis and absent mitotic figures (Fig. 1c and d). Immunohistochemically, the neoplastic cells had faint cytoplasmic and membranous reactivity for CD3 and lacked CD79a and granzyme B immunoreactivity (Fig. 1e and f), confirming the diagnosis of TZL. A lymph node aspirate submitted for flow cytometry showed that lymphocytes were highly positive with antibodies to CD5 and MHC II, while detection of CD3 and CD45 was dim and absent, respectively [12] (Fig. 1g). Abdominal ultrasound findings were unremarkable, and MRI of the brain showed approximately 50% reduction in size of the intracranial lesion. Due to the indolent nature of TZL and the patient being well, monitoring without specific therapy was recommended.

Nine months following the diagnosis of TZL, the patient was presented again due to lethargy and dullness, and mild to moderate generalized peripheral lymphadenopathy was noted. The results of a CBC and serum biochemistry analysis were again unremarkable, and an MRI of the brain was performed. Mild obstructive hydrocephalus attributed to an intramedullary lesion in the cranial cervical spine with meningeal enhancement was noted. There was no apparent progression of the previously identified intracranial neoplasm. Therapy with prednisone (1 mg/kg q24h) was initiated with the intent to alleviate edema and hydrocephalus. The dog’s lethargy and dullness improved; however, reduction in prednisone dosage over the subsequent weeks resulted in recurrent malaise. Therefore, a maintenance prednisone dosage of 0.7 mg/kg q48h was prescribed.

Two months later, the owner noted progressive peripheral lymphadenomegaly, which was confirmed during physical examination at the teaching hospital. The marked increase in lymph node size was interpreted as progression of the TZL, and chemotherapy with chlorambucil (4 mg/m^2^ q24h) was added to prednisone. Three weeks later, the owners reported that an episode of pain and weakness had occurred at home, which prompted an increase in prednisone dosage to 1 mg/kg q24h. The owners noted no further episodes.

**Fig. 2:**
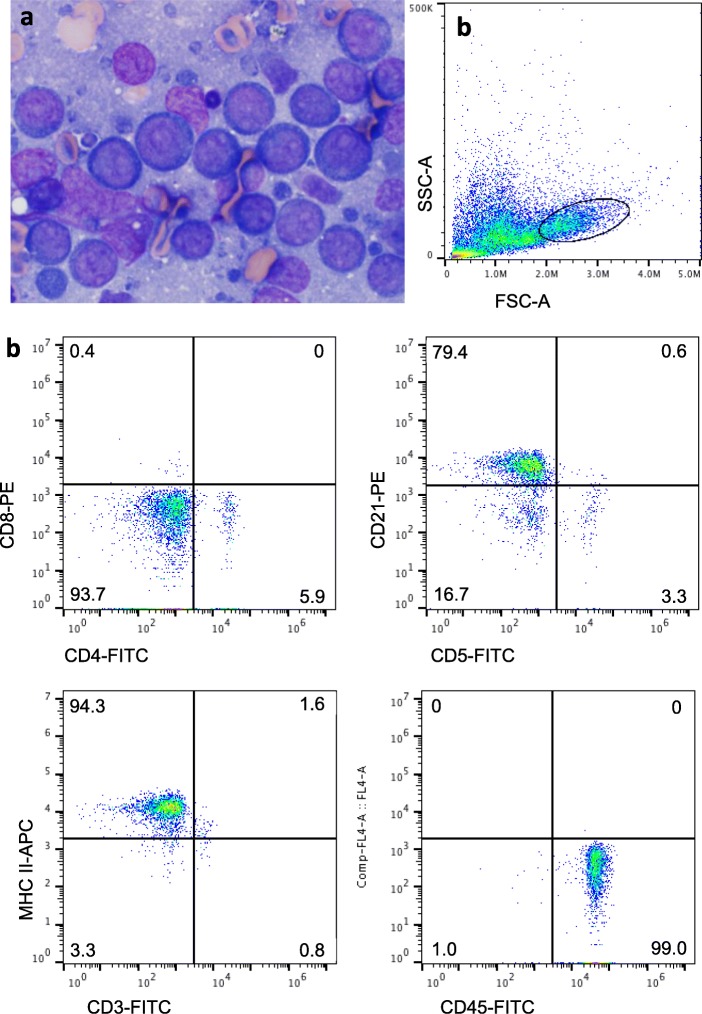
Popliteal lymph node aspirate 13 months after sample in Fig. 1 was obtained. **a** The lymphocytes are large and round with intensely basophilic cytoplasm, and prominent, mostly single, large nucleoli. **b** On flow cytometry, a prominent population of large lymphocytes is apparent, and these lymphocytes are highly positive for CD21, MHC II and CD45. These findings are typical of DLBCL

At a recheck assessment 6 weeks following the start of chlorambucil therapy, lymphadenomegaly was unchanged. The dosage of chlorambucil was increased to 20 mg/m^2^ q14d. Eleven days later the dog was re-admitted due to hyporexia, lethargy and progressive lymphadenomegaly. Moderate neutrophilia with mild left shift and biochemical abnormalities consistent with a steroid hepatopathy were noted on bloodwork (Additional file 1: Table S1), and abdominal ultrasound revealed medial iliac and inguinal lymphadenomegaly, and diffuse nodular hepatopathy. Cytologic evaluation of a prescapular lymph node aspirates showed a homogeneous population of large lymphocytes with intensely basophilic cytoplasm, central to slightly eccentric nuclei and indistinct paranuclear clear zones, which was interpreted as a large cell lymphoma (Fig. 2a). On flow cytometry, the cells were highly positive for CD21, CD45 and MHC II and negative for CD3, CD4, CD5 and CD8, consistent with a diagnosis of LBCL (Fig. 2b). There was also a population of small lymphocytes, and these lymphocytes expressed a similar constellation of antigens as those of the prior TZL (data not shown). The dog was hospitalized and 10,000 IU of L-asparaginase, 0.2 mg/kg of dexamethasone and 0.7 mg/m^2^ of vincristine were administered. Lymph node size decreased within 24 h, but the owners elected not to continue with further therapy, and no additional anti-neoplastic therapy was instituted. The dog was euthanized by intravenous pentobarbital injection at the primary veterinary clinic 2 months following the diagnosis of LBCL (15 months after diagnosis of TZL) due to progressive lethargy, weakness, and lymphadenomegaly. An necropsy was not performed.

**Fig. 3:**
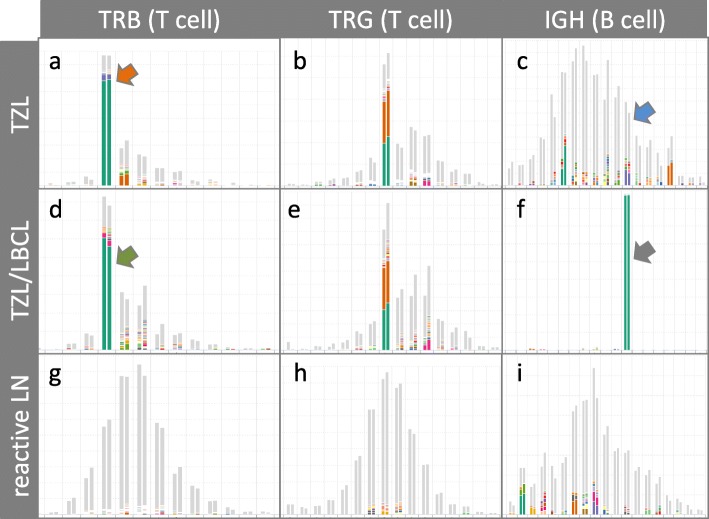
Sequencing-based clonality test results, analysed and visualized by ARResT/Interrogate (http://arrest.tools/interrogate), from replicate samples diagnosed as TZL (**a**-**c**), ‘composite’ TZL and LBCL (**d**-**f**), and from a reactive lymph node of a different patient (**g**-**i**). X-axis: amino acid length of junctional region, double bars per junction length represent replicates; Y-axis: abundance of clones. The 100 most abundant clones are displayed as colored slices of a bar, less abundant clones are grey. Identical colors within one subfigure represent identical clonotypes. Dominant clones in a polyclonal background are visible for the TRB and TRG loci in the TZL sample, and in the TZL/LBCL sample. For the IGH locus, polyclonal results were obtained for the TZL sample and a clonal result for the TZL/LBCL sample. The reactive lymph node control sample gave polyclonal results for all loci (**g**-**i**). Bars with colored arrows are highlighted in Fig. 4

**Fig. 4:**
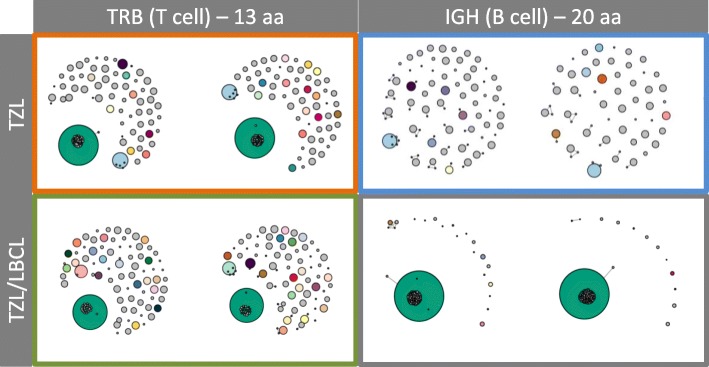
Network diagrams reflecting the junctional sequence homology of the most abundant clones with a given junctional length (TRB: 13 amino acids; IGH: 20 amino acids) for the TZL (top row) and the TZL/LBCL (bottom row) in replicate. Bubble size corresponds to read abundance. Smaller bubbles superimposed on dominant clones represent clones with similar sequence and likely reflect true biological sequence variation as well as PCR and sequencing errors. Border colors correspond to the arrow colors in Fig. 3

To investigate a potential relationship between the initial TZL and subsequent LBCL, NGS-based clonality testing was performed after euthanasia using DNA extracted from lymph node samples collected at the time of diagnosis of each lymphoma (Figs. 3 and 4; Additional file 2: Table S2). The initial sample diagnosed as TZL yielded polyclonal results with primers targeting the immunoglobulin heavy chain (IGH) locus (Fig. 3c), and clonal rearrangements in a polyclonal background with primers targeting the T cell receptor gamma (TRG) (Fig. 3b) and T cell receptor beta (TRB) (Fig. 3a) loci. The dominant TRB clone was productive and utilized the genes TRBV16/TRBJ2–1. The TRG repertoire contained two dominant clones of approximately equal abundance, both of which were unproductive and utilized TRGV2/TRGJ3–2. The subsequent sample from the time of LBCL diagnosis yielded virtually identical results for the T cell loci compared to the initial sample. The same TRB and TRG clones were present in a polyclonal background (Fig. 3d-e), suggesting that the TZL had persisted. In contrast to the initial sample, the second sample gave a clear-cut clonal result for the IGH locus (Fig. 3f). The dominant clone was productive and utilized genes IGHV3–6/IGHJ3. No dominant clones were seen for any loci in the lymph node control (Fig. 3g-i).

## Discussion and conclusions

This report describes the emergence of LBCL in a dog with pre-existent and persistent TZL. In human oncology, presence of two distinct, clonally unrelated, lymphomas within the same organ is termed “composite lymphoma”. This entity comprises < 5% of all lymphomas in humans [13], but has not previously been reported in dogs. The diagnosis of composite lymphoma requires morphological, immunohistochemical, and molecular assessment [13]. In the case reported here, this diagnosis was based on the dissimilar cytomorphological and immunophenotypic features of the tumors as well as the two distinct clonal signatures determined by NGS-based clonality testing. Transformation of indolent hematologic neoplasms into more aggressive forms, such as chronic lymphocytic leukemia into high-grade lymphoma, has previously been reported, and appears to occur occasionally in dogs [14–16]. However, in this case, the combined evidence of multiple testing modalities strongly suggests the concurrence of two distinct lymphomas rather than evolution of an indolent lymphoma into a more aggressive variant.

Differentiating relapse from development of a de novo tumor is challenging for most cancer types. Lymphoid cancers differ in this regard because every lymphocyte clone carries a unique DNA sequence that can be used as a genetic fingerprint to track lymphocyte clones over time and across anatomic sites. This unique gene sequence is generated early in lymphocyte development by rearrangement of antigen receptor genes, and confers every lymphocyte clone with unique antigen specificity. Clonality testing assesses the diversity of lymphocyte antigen receptor genes in a given lymphocyte population. In the initial sample, clonality testing confirmed the diagnosis of TZL based on the presence of clonal TRB and TRG rearrangements. A single productive TRB rearrangement and two unproductive TRG rearrangements were consistent with an alpha/beta T cell lineage of the neoplastic clone, and usage of TRGV2/TRJ3–2 by both dominant clones was suggestive of a bi-allelic rearrangement rather than the rearrangement of two different cassettes on the same chromosome. The same rearrangements were found in the second sample at similar abundance, which suggests persistence of the neoplastic T cell clone in the face of treatment. In addition to the clonal TRB and TRG rearrangements, the second sample showed a dominant IGH clone that comprised about 88% of all rearrangements. This finding not only confirms the diagnosis of a B cell lymphoma but also suggests that the B cell lymphoma is a de novo tumor rather than a progression of the TZL with altered immunophenotype. In contrast to cell surface marker expression, which can be influenced by micro-environmental stimuli and a cell's developmental stage and viability, lymphocyte antigen receptor gene rearrangements are stable throughout the life of a lymphocyte [17]. Consequently, if the B cell lymphoma had been a transformed progression of the previously diagnosed TZL, then the dominant IGH clone detected in the second sample would have had to be present in the initial sample. However, the initial sample had a diverse polyclonal B cell repertoire, and the sequence of the clonal IGH rearrangement was not detected in the initial sample.

The use of sequencing-based clonality testing provided a distinct advantage over electrophoresis-based methods. Traditionally, clonality testing utilizes gel electrophoresis to visualize the diversity of lymphocyte antigen receptor gene arrangements in a given sample. Since this method only distinguishes antigen receptor genes by size, it can result in equivocal results when the signal of a neoplastic clone is quenched by noise of non-neoplastic lymphocytes. Sequencing-based clonality testing yields a higher ‘clonal resolution’ because it can distinguish lymphocyte clones based on sequence on top of size [18, 19]. In this study, sequencing-based testing readily identified TRB and TRG clones in both samples despite the presence of a polyclonal background. Furthermore, identification of the TRB and TRG gene sequences of the neoplastic clone unequivocally determined that the dominant T cell clone was identical in both samples. Identifying clones by gene sequence confers a higher confidence in both clones being identical than if clones are identified by size only. Another advantage of NGS-based clonality testing is that once the sequence of a neoplastic clone has been determined, it can be traced in samples even if it comprises a minute fraction of all rearrangements [10]. In the current case, identification of the IGH gene sequence of the LBCL in the second sample allowed searching for this ‘index sequence’ in the initial sample. The fact that the IGH index sequence could not be found in the initial sample strongly suggests that this B cell clone was not present at the time when the TZL was initially diagnosed. Of note, the sensitivity of detecting an index clone is highly dependent on the sequencing depth.

Although NGS was useful to identify the presence of two distinct clones in this case, other diagnostic approaches were also necessary. An entire affected lymph node was initially removed and assessed histopathologically and immunohistochemically to confirm the cytological diagnosis of TZL. Sections consisted of a homogeneous population of small lymphocytes with rare remnant follicles. Cytologic evaluation of an aspirate of the contralateral popliteal lymph node collected 1 year after the initial sample identified cells morphologically consistent with LBCL rather than TZL, which prompted immunophenotypic assessment. Flow cytometry confirmed LBCL, which in virtually all cases in dogs is a DLBCL [4]. Although histopathological assessment of the affected lymph node was not performed, the flow cytometric findings of CD21, CD45 and MHC II positivity combined with large cell size were highly consistent with the diagnosis of DLBCL [20]. Among lymphomas in dogs, TZL is a unique entity since even without therapy the neoplasm may not progress at all or only slowly; there is a strong breed predilection; the pan-leukocyte antigen CD45 is typically undetectable on tumor cells; and the B cell antigen CD21 may be present at low level [6]. Furthermore, cells with this immunophenotype have been identified in older Golden Retriever dogs without evidence of lymphoid neoplasia, and some of these dogs also were reported to have clonal T cell populations [21]. Of note, the resolution of clonal peaks with electrophoresis-based methods is lower than with sequencing-based methods, and it is conceivable that clonal populations identified by electrophoresis may be more diverse if assessed by sequencing. Therefore, many aspects of the biology of TZL remain incompletely characterized.

It is possible that the dog in this report had an increased risk of developing secondary neoplasms following radiation of the brain tumor. Radiation therapy induces a multitude of adverse effects, including systemic effects from local therapy [22]. Such effects can compromise the immune system, which in turn might reduce immune surveillance and increase the risk of subsequent cancer development. While in humans tumor irradiation is most often associated with secondary myeloid neoplasms, similar associations in dogs are undetermined [23]. In general, knowledge of genetic lesions underlying lymphomagenesis in dogs is sparse, and a limited set of mutations was more highly associated with breed than with lymphoma type [24].

Treatment and prognosis of composite lymphoma in humans varies depending on histological subtype [13]. Canine TZL has an indolent disease course, but DLBCL is an aggressive lymphoma with median progression free survival of 251–252 days, when treated with combination chemotherapy [5, 25]. Induction therapy for the dog in this report was a standard dose of L-asparaginase and vincristine, but a durable clinical response was not expected due to the prior long-term exposure to glucocorticoids [26]. Unfortunately, despite an initial favorable response, therapy was not continued, and outcome could not be fully assessed.

The patient had been diagnosed with an intracranial mass most consistent with glioma several months prior to the first diagnosis of lymphoma. Without histopathologic evaluation, non-neoplastic causes of brain masses such as vascular events or granulomatous inflammation cannot be entirely ruled out. However, the clinical features and MRI characteristics of intra-axial location, T2/FLAIR hyperintensity, T1 hypointensity, lack of contrast enhancement, and mass effect, were most suggestive of a neoplasm such as a low-grade glioma [27–29]. Definitive radiation therapy resulted in at least 16 months of objective tumor response in this patient, which is similar or slightly longer than reported for other intra-axial tumors [30, 31].

Limitations to this investigation were the unavailability of a biopsy from the lymph node with concurrent TZL/LBCL, and lack of post mortem assessment. Histopathology and immunohistochemistry of the second lymphoma would have allowed more definitive diagnosis of LBCL, and illustrated morphological findings associated with concurrent TZL and LBCL. Similarly, post mortem evaluation would have allowed conclusive identification of the brain lesion and the extent of the lymphoma. Nevertheless, evidence for a composite lymphoma in this case was considered very strong based on multiple sophisticated and complementary diagnostic approaches.

In conclusion, this report of a composite lymphoma in a dog highlights the value of multiple diagnostic approaches to differentiate between two de novo lymphomas rather than transformation of a single clone.

## Supplementary information

**Additional file 1:** **Table S1.** Results of complete blood count and serum biochemistry.

**Additional file 2:** **Table S2.** Primer Sequences.

**Additional file 3.** Materials and Methods.

## Data Availability

Supporting data (Additional file 1: Table S1; sequential CBC and biochemistry results, Additional file 2: Table S2; primer sequences, Additional file 3; DNA extraction and PCR/sequencing protocols) are included as an additional file to this article. The sequencing data are available under the BioProject ID PRJNA542543. Additional data are available from the corresponding author upon request.

## Abbreviations

CBC: Complete blood count
DLBCL: Diffuse large B cell lymphoma
LBCL: Large B cell lymphoma
MRI: Magnetic resonance imaging
NGS: Next generation sequencing
PARR: PCR for antigen receptor gene rearrangement
PCR: Polymerase chain reaction
TCR: T cell receptor
TZL: T zone lymphoma
