# Supplementary material for: Composite lymphoma of concurrent T zone lymphoma and large cell B cell lymphoma in a dog
Source: BMC Vet Res. 2019 Nov 16;15:413. doi: 10.1186/s12917-019-2154-8 (PMC6858704; doi:10.1186/s12917-019-2154-8)
Supplement: Supplementary file 3 — Additional file 3. Materials and Methods. [file 12917_2019_2154_MOESM3_ESM.docx]

**Material and Methods**

**DNA extraction and PCR**

DNA was extracted from the left popliteal lymph node (2016, TZL, frozen tissue) and right popliteal lymph node (2017, TZL/DLBCL, unstained cytology slide) using the QIAamp DNA Micro Kit (Qiagen, Valencia, CA). DNA extracted from a frozen, submandibular lymph node of a healthy dog was used as polyclonal control and molecular biology grade water was used as no template control. The extracted DNA was quantified by spectrophotometry (Nanodrop, ThermoFisher, Waltham, MA) and normalized to 10ng/uL. The rearranged canine immunoglobulin heavy-chain (IGH) genes, the canine T cell receptor beta (TRB) genes and the T cell receptor gamma (TRG) genes were amplified by multiplex PCR. The IGH primer set has been described previously (Hwang et al., 2019), the TRB and TRG primer sequences are given in supplemental table 2. PCR was performed using the NEB Q5 high-fidelity polymerase in replicate according to the manufacturer’s instructions and visualized by capillary electrophoresis (Qiagen, Valencia, CA; high resolution cartridge, OL 500; QIAxcel ScreenGel Software). All antigen receptor loci were amplified in separate reactions and pooled by replicate and sample after successful visualization.

**Library preparation, sequencing and bioinformatics analysis**

Products were cleaned-up through a modified solid-phase reversible immobilization bead protocol using Mag-Bind RXNpure Plus beads (Omega Bio-Tek, Norcross, GA). End prep and adapter ligation were performed using the NEBNext Ultra II DNA Library Prep Kit for Illumina (E7645; New England Biolab, Ipswich, MA) according to the manufacturer’s instructions. Subsequently, samples were fluorometrically quantified (Qubit dsDNA HS Assay Kit and Qubit 2.0 Fluorometer; Invitrogen, Carlsbad, CA) and normalized to 4nM prior to final pooling.

The library was sequenced on an Illumina MiSeq instrument in-house using the v2 PE300 reagent kit (Illumina, San Diego, CA). The library was loaded at a concentration of 10pM with 10% 12.5pM PhiX by volume. Sequence demultiplexing and retrieval were done through Illumina BaseSpace. Subsequent quality trimming and filtering were done using the Trimmomatic software (PHRED 30, length 50 bp). Antigen receptor gene specific analyses were done using the ARResT/Interrogate software (<http://arrest.tools/interrogate>; Bystry et al., 2017) and custom R scripts. All raw sequencing data are available through the Sequence Read Archive BioProject accession PRJNA542543.

**References**

[Bolger AM](https://www.ncbi.nlm.nih.gov/pubmed/?term=Bolger%20AM%5BAuthor%5D&cauthor=true&cauthor_uid=24695404), [Lohse M](https://www.ncbi.nlm.nih.gov/pubmed/?term=Lohse%20M%5BAuthor%5D&cauthor=true&cauthor_uid=24695404), [Usadel B](https://www.ncbi.nlm.nih.gov/pubmed/?term=Usadel%20B%5BAuthor%5D&cauthor=true&cauthor_uid=24695404). Trimmomatic: a flexible trimmer for Illumina sequence data**.** [Bioinformatics.](https://www.ncbi.nlm.nih.gov/pubmed/24695404) 2014 Aug 1;30(15):2114-20

Bystry, V., Reigl, T., Krejci, A., Demko, M., Hanakova, B., Grioni, A., Knecht, H., Schlitt, M., Dreger, P., Sellner, L., Herrmann, D., Pingeon, M., Boudjoghra, M., Rijntjes, J., Pott, C., Langerak, A.W., Groenen, P.J.T.A., Davi, F., Bruggemann, M., Darzentas, N., 2017. ARResT/Interrogate: an interactive immunoprofiler for IG/TR NGS data. Bioinformatics 33, 435–437.

Hwang, M.-H., Darzentas, N., Bienzle, D., Moore, P.F., Guscetti, F., Morrison, J., Keller, S.M., 2019. A review of canine B cell clonality assays and primer set optimization using large-scale repertoire data. Vet. Immunol. Immunopathol. 209, 45–52.
